# Power Enhancement and Spot Homogenization Design for Arrayed Semiconductor Lasers

**DOI:** 10.3390/mi15060744

**Published:** 2024-05-31

**Authors:** Shunshun Zhong, Jun Xiong, Cong Xu, Fan Zhang, Ji’an Duan

**Affiliations:** 1 State Key Laboratory of Precision Manufacturing for Extreme Service Performance, College of Mechanical and Electrical Engineering, Central South University, Changsha 410083, China; 2 Aviation Machinery Manufacturing College, Changsha Aeronautical Vocational and Technical College, Changsha 410083, China; 3 School of Automation, Central South University, Changsha 410083, China

**Keywords:** fiber pump lasers, fiber coupling, ray tracing, coupling tolerance

## Abstract

Improving the spot brightness and uniformity of arrangement of the array laser is conducive to ensuring the beam quality of the fiber laser. Based on the light tracing principle, the optical model performance of two common fiber lasers was first analyzed. Then, a novel rotationally polarized optical model with high power and spot uniformity was designed and optimized on the basis of the aforementioned analysis. The results of the evaluation metrics of the multi-indicator optical model show that the spot uniformity of our proposed model improved by 24.03%, the power improved by 0.55%, and the maximum light distance was shortened from 120 mm to 82.58 mm. Further, the results of the coupling tolerance analysis of the optical elements show that the total coupling efficiency was 89.04%. The coupling power and tolerance relationships did not produce degradation compared with the traditional model. Extensive comparative results show that the designed rotationally polarized optical path model can effectively improve the optical coupling efficiency and spot uniformity of arrayed semiconductor lasers.

## 1. Introduction

Fiber pump lasers have been widely applied in the fields of industrial processing, country defense, and medicine aesthetics due to high power and spot homogenization [1,2,3,4]. For a typical semiconductor laser chip, the large difference between its fast-axis divergence angle (20~60°) and slow-axis divergence angle (6~20°) results in a low proportion of the beam focused into the fiber, which affects the optical performance of the laser [5,6]. To increase the power of the fiber laser, in addition to beam collimation of the fast and slow axes of the diode laser, the power of the fiber laser can be multiplied by increasing the number of diode lasers [7,8,9]. Since the introduction of the fiber laser, many researchers have worked to improve its coupling efficiency and beam uniformity the using beam-shaping technique.

Beam-shaping technology mainly utilizes reflection and refraction techniques to split and rearrange the beam so that the beam is homogenized in the fast- and slow-axis directions. In 2012, Ghasemi achieved beam shaping using a V-stack reflector and a polarized beam combination element, improving the beam symmetry and power density [10]. Yu et al. proposed a compact beam-shaping system with two 45° parallelogram plates and a prism array to improve the beam quality in 2015 [11]. In 2016, Xiong et al. proposed a design method for a slow-axis collimated lens with a variable radius of curvature of a semiconductor laser bar based on Snell’s law and phase constancy in front of the optical field and achieved a divergence angle of 6 mrad for a slow-axis collimated beam [12]. In 2019, Yan et al. analyzed the relationship between the positional error of each original element in high-power semiconductor lasers and the beam-forming effect, providing theoretical guidance for the packaging of high-power lasers [13]. Yu et al. designed a simple beam-shaping system utilizing trapezoidal prisms and a set of parallelogram plates to improve beam symmetry and reduce linewidth, achieving high-performance results with an output power of 272 W and a luminous efficiency of 85% [14]. In 2021, Lou et al. combined 40 collimated Ld’s and coupled them into an optical fiber with a core diameter of 200 μm/NA = 0.22, achieving a coupling efficiency of up to 94% and a total output power of 27.6 W [15]. However, traditional beam-shaping methods only consider improving the optical coupling efficiency and do not give further thought to beam uniformity, resulting in uneven spot shapes after shaping, which affects the overall performance of the fiber laser.

In this paper, a novel fiber laser model is designed from the consideration of improving both fiber coupling efficiency and spot shape homogenization. The main contribution of this paper consists of the following:

- A rotationally polarized optical model that balances high optical coupling efficiency and high spot uniformity is designed to improve the comprehensive performance of arrayed semiconductor lasers;
- The spot uniformity parameter is introduced, and the new optical model of the array laser is compared to the traditional model. The results show that its spot uniformity was improved by 24.03%, and the optical coupling efficiency reached 89.04%;
- The relationship between the positional deviation of key optical components and the coupling efficiency in the novel optical model was analyzed, which provides reliable theoretical guidance for the subsequent packaging of fiber lasers.

## 2. Theory Analysis and Optical Path Design

### 2.1. Beam Shaping and Quality

**Figure 1:**
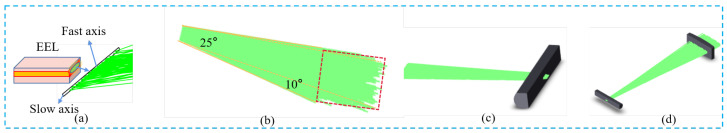
(**a**) EEL; (**b**) the initial divergent beam of a laser array beam; (**c**) laser beam after FAC collimation; (**d**) laser beam after FAC and SAC collimation.

In fiber pump lasers, we used edge-emitting laser (EEL) chips, so beam shaping was required to obtain higher power and beam quality. The EEL used in this study had a luminescence size of 5 μm × 100 μm, its fast-axis divergence angle was 25°, and its slow-axis divergence angle was 10°. We used this chip to form an array laser to realize the multiplication of the light output power. The laser chip energy of each linear array was designed at 10 W. The beam-shaping theory can be expressed in terms of the ABCD transmission matrix as in Equation (1) [16]:

(1) $$\begin{array}{c} q_{2}=\frac{Aq_{1}+B}{Cq_{1}+D},\\ \frac{1}{q_{1}}=\frac{1}{R}-\frac{i\lambda }{\pi \omega^{2}}, \end{array}$$

where $q_{1}$ and $q_{2}$ are the radii of the complex curvature of Gaussian beams before and after beam shaping, respectively. ABCD is the parameter of the optical element matrix, which can be selected differently depending on the element. R represents the radius of curvature of the Gaussian beam. ω and λ represent the Gaussian beam waist size and transmission wavelength of 905 nm, respectively. The beam parameters of any Gaussian beam can be obtained after crossing different optical elements based on Equation (1). In order to observe the beam shaped by the optical elements more intuitively, we used ZEMAX software version 2016.5 based on the principle of ray tracing, and the results of the beam emitted by the EEL passing through the fast axis collimating lens (FAC) and slow-axis collimating lens (SAC), respectively, are shown in turn in Figure 1.

Figure 1a,b shows the side-emitting laser model and its initial divergent beam scenario, respectively. Its fast axis diverged to 25°, while its slow axis diverged to 10°, and Figure 1c shows that after the action of the FAC lens, the divergence angle in the fast axis direction became very small, only about 0.043 mrad after calculation. The beam was still divergent in the slow axis. Therefore, it can be seen from Figure 1c that after passing through the SAC lens, the divergence angle of the beam in the slow-axis direction also decreased from 87.5 mrad to 0.099 mrad. In addition, from the above analysis, it is shown that FAC and SAC acted to collimate the beam in the fast and slow axes of the laser, respectively.

**Figure 2:**
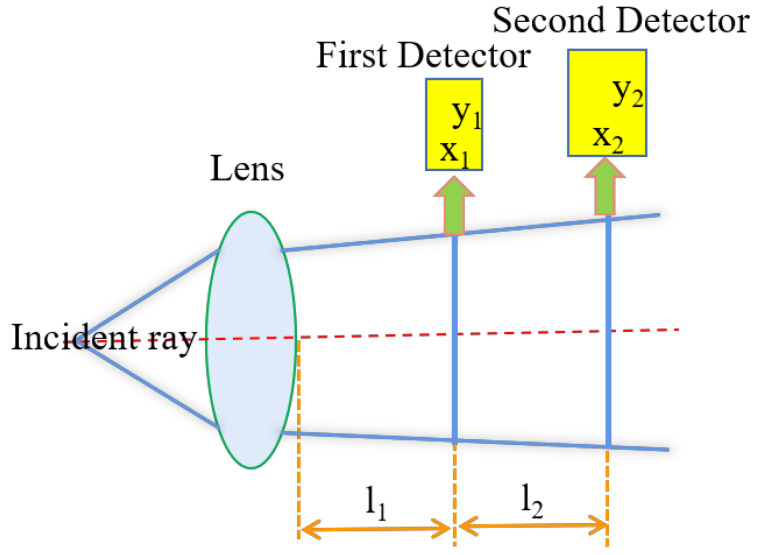
Principle of divergence angle calculation.

The methods commonly used to evaluate the beam quality are original diffraction limit multiple factors, energy ratio of the circumference, M^2^ factor or its reciprocal K factor (beam transmission factor). The definition of various beam quality parameters corresponding to different application purposes reflects the fact that the beam quality of the emphasis is different [17,18,19]. In general, we used the beam parameter product (BPP) to estimate the beam quality of a laser diode [19], which is defined as follows:

(2) $$\mathrm{BPP}=\omega_{0}\times \theta ,$$

where $\omega_{0}$ is the beam waist radius, and θ is the far-field divergence angle. In this way, we took half of the size of the light source as the beam waist radius and half of the size of the divergence angle as the far-field beam divergence angle. Then, two rectangular detectors were set at 10 mm and 100 mm from the lens. The divergence angle was calculated as shown in Figure 2.

As is shown in Figure 2, two rectangular detectors at the rear end of the lens were arranged, where $l_{1}$ is the horizontal distance between the first detector and the lens, and $l_{2}$ is the horizontal distance between the two detectors. x1 and x2 represent the fast axis distribution sizes on the two detectors, respectively. y1 and y2 represent the slow axis distribution sizes on the two detectors, respectively. As a result, ω and Φ can be calculated using the following Equations (3)–(6):

(3) $$\omega_{1}=x/2$$

(4) $$\omega_{2}=y/2$$

(5) $$\varphi_{1}={(y}_{2}-y_{1})/2l_{2}$$

(6) $$\varphi_{2}={(x}_{2}-x_{1})/2l_{2}$$

The divergence angle was effectively compressed after the fast-axis beam and slow-axis beam were collimated separately using the FAC and SAC designed in this paper. It can be obtained that the BPP in the direction of the fast axis reached 0.592 mm·mrad, while the BPP in the direction of the slow axis was 8.242 mm·mrad. Specific calculation data are shown in Table 1.

In general, the light beam collimated by fast and slow collimators appeared as a rectangle with a large, slow-axis size and a small, fast-axis size in the far field. Thus, the array of semiconductor lasers was arranged along the fast axis so that the combined spot was close to a square shape. The number of light sources allowed to be aligned along the fast axis depends on two aspects. On the one hand, it depends on the height difference between the luminous points, i.e., the geometric dimension. The smaller the height difference, the more can be stacked and the more demanding the workmanship. On the other hand, the product of fiber beam parameters will also limit the number of arrays. The product of optical fiber BPP can be expressed by the following Equations (7)–(10):

(7) $$BPP_{fiber}=\frac{D_{fiber}.NA}{2},$$

(8) $${NA}_{max}=\mathrm{Sin}\theta_{max}=\frac{1}{n_{0}}\sqrt{n_{1}^{2}-n_{2}^{2}},$$

(9) $$BPP_{\mathrm{fiber}}=\omega \theta =\frac{R}{2}\sin\alpha =\frac{R}{2}*NA,$$

(10) $$n_{max}=\frac{\sqrt{{BPP}_{fiber}^{2}-{BPP}_{slow}^{2}}}{BPP_{fast}},$$

where $D_{fiber}$ is the optical fiber’s inner diameter, and NA is the numerical aperture. The fiber uses N-BK7 as the core material and N-LLF6 as the cladding material, with refractive indices of 1.5168 and 1.5317, respectively. The calculation of its numerical aperture can be substituted into Equation (8), where $n_{0}$ is the refractive index of light in air. n1 and n2 represent the refractive index of light in the inner and outer diameters of the fiber. The BPP of optical fiber can be calculated with 100/2 ∗ 0.2131 = 10.655 mm·mrad. The number of laser chips that can be arrayed in the fast axis direction is limited by Equation (10). It was calculated that we could array up to 11 laser chips on the fast axis. We designed the optical model with a multimode fiber core diameter of 100 μm.

### 2.2. Optical Model Design and Analysis

**Figure 3:**
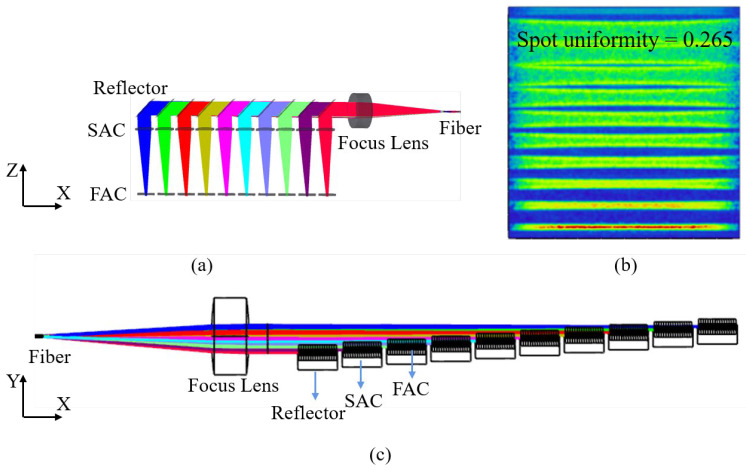
Ten laser chips. (**a**) Non-polarization path model in X-Z direction; (**b**) beam image before focusing; (**c**) non-polarization path model in X-Y direction.

In order to avoid reaching the limit value, we arranged 10 laser chips on the fast axis. The vertical (Y) distance between light sources was 0.3 mm, and the horizontal distance (X) was 4.742, as shown in Figure 3c. Theoretically, the smaller the value, the more uniform the distribution of light spots. However, since the beam still has a certain divergence angle after collimation, when the optical path is far away, too small a distance will cause part of the beam to be blocked by the reflector, resulting in a reduction in optical power. An obvious phenomenon can be seen in Figure 3b; the light spot diverges more and more from bottom to top, which also verifies the existence of divergence angle after collimation. Due to the presence of the laser array, the 10-channel beam must lead to optical length differences. Reducing the uneven light distribution caused by optical path difference is also one of the problems to be solved.

**Figure 4:**
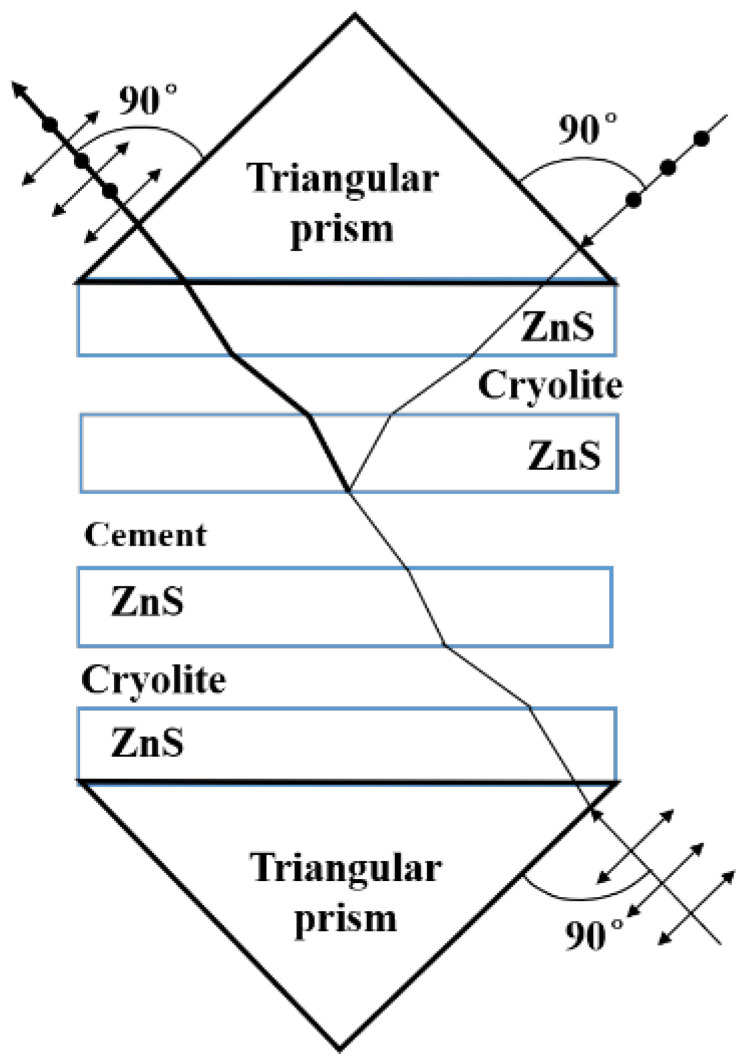
Schematic diagram of a polarizing beam splitter.

Increasing power by only increasing the number of beams in the same direction seems impossible due to limitations in space distance and fiber size. When the number of beams in one direction reaches its limit, an additional laser light path can be added through the use of polarization combinations, and then beam-combining techniques can be used to multiply the output power of the fiber [20]. A schematic diagram of a polarizing beam splitter is shown in Figure 4. It contains two triangular prisms and coats the prisms with a special thin film layer. In the picture, the special film layer is composed of ZnS, cryolite, cement, etc. Light with different polarization states will have different reactions when passing through the film, in which the P-polarized light is refracted several times and is emitted from a lens parallel to the direction of incidence [21,22,23]. The S-polarized light will be emitted perpendicularly to the direction of incidence after being fully reflected in the ZnS and CEMENT films. Using this principle and the principle that the optical path is reversible, the polarization beam-combining technique can be applied to the high-power semiconductor laser.

**Figure 5:**
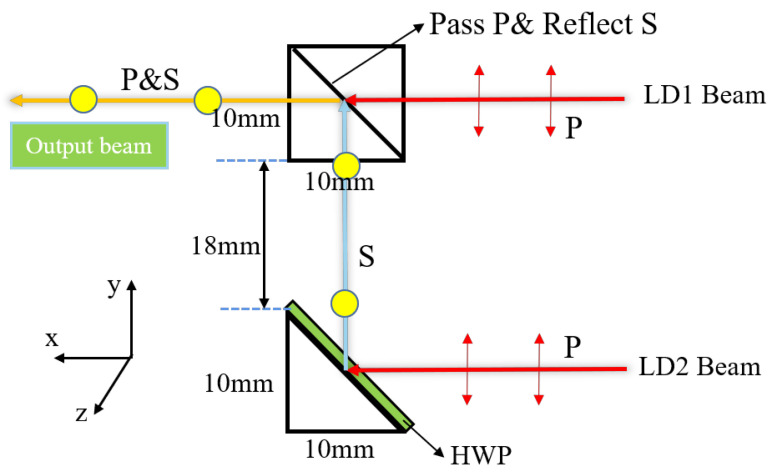
The structure, size, and orientation of a polarized beam synthesizer.

The structure and optical configuration of the polarization beam combiner is shown in Figure 5. The polarized beam synthesizer consists of a cubic prism and a triangular prism with a bonded half-wave plate (HWP). The polarization film (pass P-polarized light) is coated in the cube prism, which can be used to realize the polarization beam combination.

The polarization state of the input LD beam is a TE wave, and the electric field oscillations of the TE wave are parallel to the slow axis, so the beam is defined as P-polarized light in Figure 5, indicated by the red line. The red ray at the bottom of the image can be deflected 90° through a triangular prism. Before the beam is deflected by 90°, the HWP can shift its polarization by 90° from the red ray representing the P-polarized light to the blue ray representing the S-polarized light. The red light at the top of the image also represents incident light in the P-polarized state, which will pass through the polarizing film in the polarizing beam-splitting prism without changing the polarization state.

With all the beams passing through the polarization-splitting prism, the exiting light becomes a beam with a P polarization state and an S polarization state combined. Theoretically speaking, the combined power will be twice that of the single polarization state beam. However, the polarization-combining prism has a certain loss of light, and its reflection efficiency, that is, the exit efficiency of S-polarized light, is 99.5%, while the transmittance efficiency of P-polarized light is 90% [24,25]. 

**Figure 6:**
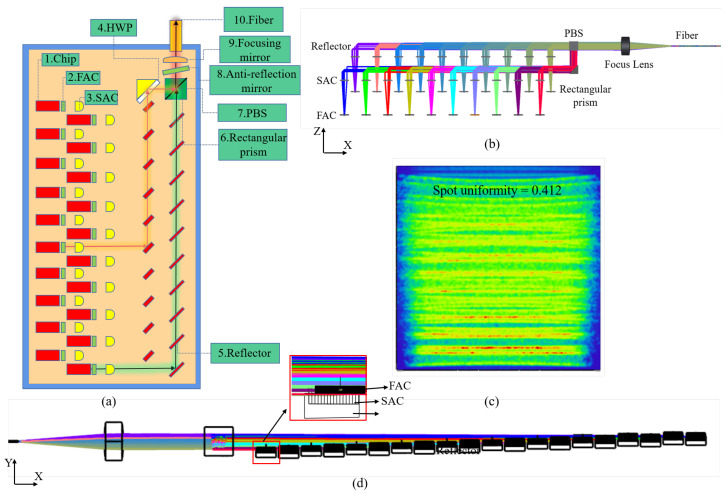
Twenty laser chips. (**a**) Polarization combined beam path model indication chart; (**b**) polarization combined beam path model in X-Z direction; (**c**) beam image before focusing; (**d**) polarization combined beam path model in X-Y direction.

Based on this beam-shaping technique and polarization beam-combining technology, we constructed a prototype for a fiber-coupled diode laser system with 20 laser chips, which is applied in Figure 6a. In order to make the optical model meet the actual condition of the lens, we set up appropriate coatings on all optical elements by applying a transmittance-enhancing layer on the surface of the lens and a high-reflective coating on the surface of the reflector. The reflectivity of optical elements is shown in Table 2.

As shown in Figure 6b,d, there was no change in the parameters of the FAC, SAC, and reflector compared with the 10-laser chip unpolarized path model, except that the incident light changed from unpolarized to P-polarized. After passing through the polarization beam combiner, the beam shape was obtained, as shown in Figure 6c. Compared with the shape of the spot of the non-polarization path model, the density of the spot obviously increased, and there was no large gap between the stripe spots. However, the uneven light distribution caused by the difference in optical paths is seen in the spot map, resulting in an isosceles trapezoidal shape of the spot distribution. It can lead to uneven beam energy entering the fiber after focusing and damage to the multimode fiber.

The beam-shaping operation mentioned above can greatly reduce the current situation of the uneven distribution of light intensity in semiconductor lasers. However, due to the divergence angle of the fast axis and slow axis and the existence of optical path difference, the shape of the spot in the far field of the non-polarized model transitions from dense to sparse from bottom to top. 

**Figure 7:**
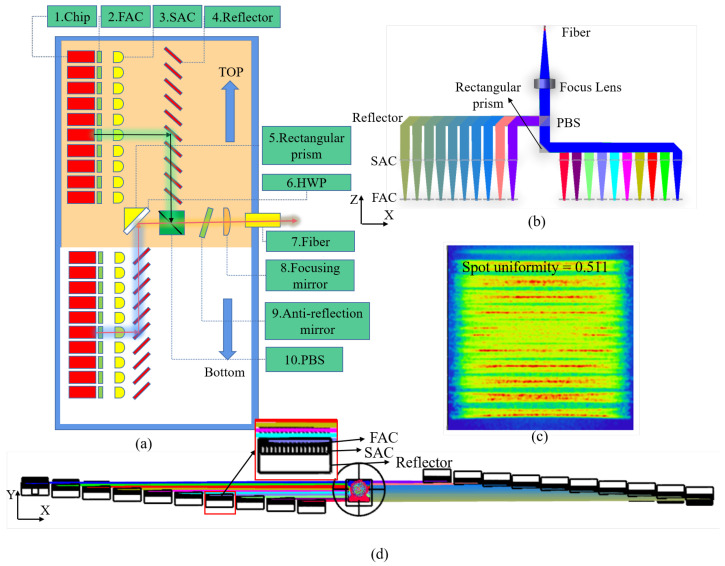
Twenty laser chips. (**a**) Rotationally polarized combined beam path model indication chart; (**b**) rotationally polarized combined beam path model in X-Z direction; (**c**) beam image before focusing; (**d**) rotationally polarized combined beam path model in X-Y direction.

Compared with the unpolarized mode, the optical model with polarized combining beams can effectively produce beam multiplication at the same position. However, due to the presence of optical length difference, the spot superposition effect of the polarization combining beam model shows long and long-length spot superposition and short and short-length spot superposition, which greatly increases the spot inhomogeneity. When the divergence angle of the fast or slow axis in the far field is large, the uneven beam distribution caused by the divergence angle is further amplified. In order to reduce this effect, we designed a reverse superposition polarized beam combination model on the basis of the existing polarized beam combination model and ultimately achieved the effect of spot homogenization through the complementarity of long- and short-length spots. The structure and spot shape are shown in Figure 7. The whole device model is distributed in steps. The blue arrows represent the ladder from low to high. The beams can converge without being blocked. The orange background indicates that the mirror will be installed in the illustrated position, and the white background indicates that the prism will be installed on the other side of the device.

Compared with the polarization combined beam path model, the new model uses the rotating polarization combined beam path model, and the separation of P-polarized light and S-polarized light can reduce the distance between adjacent beams, thus reducing the optical length difference. In addition, as shown in Figure 7a, the light source arrangement of P-polarized light is low on the left and high on the right, and the light source arrangement of S-polarized light is also low on the left and high on the right. Therefore, the superposition of light spots is dense light spots superimposed on sparse light spots, which can achieve the effect of homogenizing light spots.

The evaluation of spot uniformity can be calculated using the following [26,27]:

(11) $$U=1-\frac{1}{\frac{1}{N}\sum_{i=1}^{N}I(x_{i},y_{i})}\sqrt{\frac{\sum_{i=1}^{N}{\left[I(x_{i},y_{i})-\frac{1}{N}\sum_{i=1}^{N}I(x_{i},y_{i})\right]}^{2}}{N}},$$

where ($x_{i},y_{i}$_i_) denotes the location of the sampling pixel on the detection plane, and *N* is the total number point. A comparison of the specific parameters of the three optical path models is shown in Table 3.

Because the power of a single luminous source was 10 W, for the non-polarized model system, the power of it in front of the focusing mirrors was 95.53 W, indicating that its efficiency was 95.53%. For the polarization combined beam path model system, its efficiency was 91.72%, indicating that the optical power was lost to a certain extent after reflection and refraction through optical prisms. In contrast, the efficiency of the rotationally polarized combined optical path model before focusing was 92.47%, indicating that the reduced optical length reduced the power loss. Comprehensive analysis showed that the rotationally symmetric model performed better in the evaluation of the final coupling power and spot uniformity, which proves the effectiveness of our designed model.

From the perspective of spot uniformity, polarization combined beam path model and rotationally polarized combined beam path model can both effectively improve spot uniformity. The method of rotating superposition can make the spots more uniform. In practice, due to the use of a rotationally symmetric combined beam path model, it will be more difficult to install.

## 3. Coupling Tolerance Analysis and Discussion

For a semiconductor laser, the tiny position shift or angle deflection of the optical prism will have a great influence on the power of the whole system. To this end, the tolerance problem between the rotationally polarized combination and polarization combination beam optic model during the actual lens installation was studied.

The polarization combination optical structure of the module is shown in Figure 6a. The semiconductor laser was located at the light source end, the integrated power of the optical fiber end was 177.22 W, and the optical efficiency was 88.6%. The rotationally polarized combination optical structure of the module is shown in Figure 7a, with an optical coupling efficiency of 89.04%.

**Figure 8:**
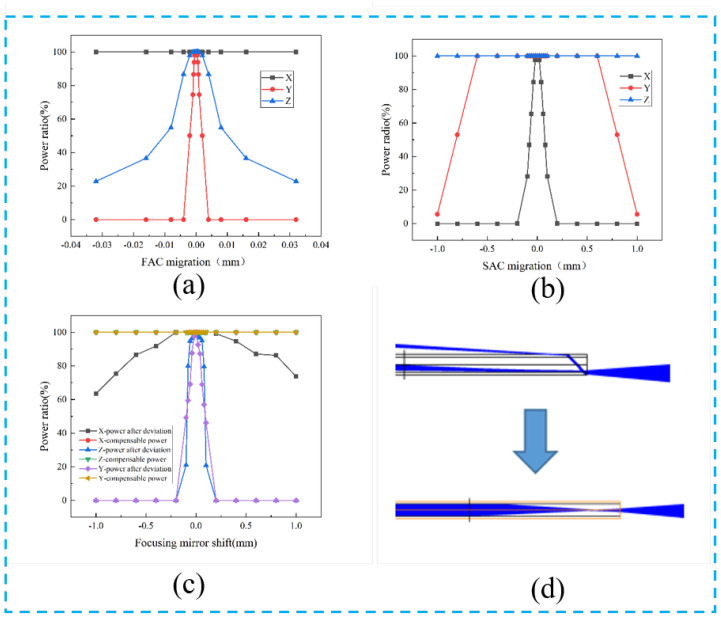
Coupling efficiency versus position curve of (**a**) FAC, (**b**) SAC, (**c**) focusing mirror, and (**d**) compensating method of focusing mirror offset.

**Figure 9:**
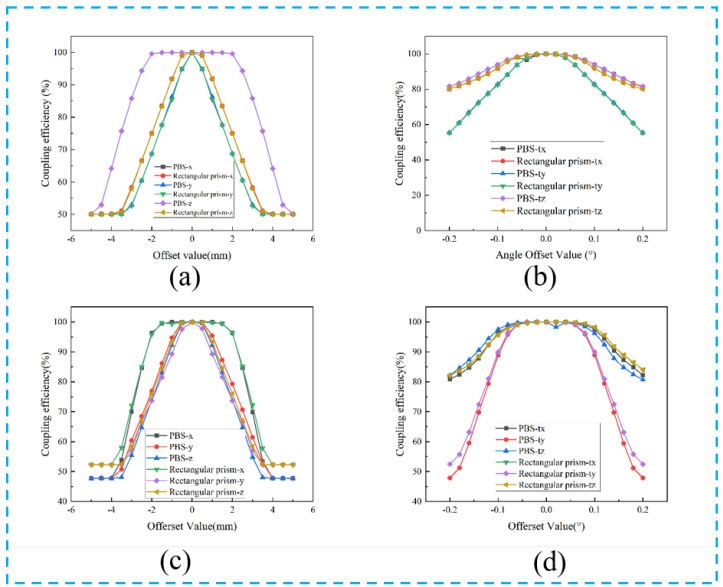
Effect of PBS and rectangular prisms position change on coupling efficiency. (**a**) x, y, and z lateral of the polarization combined beam optic model; (**b**) tx, ty, and tz angle of the polarization combined beam optic model; (**c**) x, y, and z lateral of the rotationally polarized combined beam optic model; (**d**) tx, ty, and tz angle of the rotationally polarized combined beam optic model.

In practical applications, in addition to the influence of the reflected transmittance of optical devices, the position deviation and angle deviation of optical devices are also important factors affecting the laser. Since the FAC and SAC collimation effects are exactly the same for the polarized and rotationally polarized beam-combining systems, we used only one model for the analysis. We analyzed the effect of the positional errors of the FAC, SAC, focus lens, and fiber on the coupling efficiency in the polarized beam combination system in Figure 8. In particular, the directions of X, Y, and Z are consistent with the directions of the arrows shown in Figure 5. In addition, we also compared the effect of spatial position variation of the polarized combined beam model and the rotationally polarized model PBS and right-angle prism on the final coupling efficiency of the optical system in Figure 9.

As shown in Figure 8a, for FAC, the allowable distance along the X direction is very large, but its power changes in the Y and Z directions were particularly sensitive, reaching the μm level. Among them, the allowable deviation in the y direction is only 0.5 μm, which is related to the construction of the FAC prism. Because the movement of the FAC in the Y-axis causes the spot to be deflected at an angle away from the SAC, the beam cannot pass through the SAC, resulting in a power ratio of 0. The allowable deviation in the Z direction is 2 μm. Therefore, high position accuracy is required when collimating a fast axis. However, for SAC, we can see from Figure 8b that the accuracy allowed is ±20 μm in the X direction and 600 μm in the y direction, and the allowable deviation in the z direction is limited only by its size. Overall, since the FACs are located at the front of the system, the effect of small positional offsets on the overall power is significant. The consequences of small shifts are difficult to adjust in subsequent optical path processes. In particular, it is emphasized that the power effects of FAC, SAC, and focusing mirror offsets are similar in the polarized combined light path model and the rotationally polarized combined light path model because there is little difference in the structure of these parts of the two models. However, due to variations in the optical path, the positional deviations of the PBS and rectangular prisms have a relatively large difference in power.

Although the focusing mirror offset, because it is located at the end of the system, may affect the power ratio differently across the three directions of the translation, certain measures can be taken to compensate for the power loss. As shown in Figure 8c, the allowable deviation in the Y direction is the smallest, which is ±5 μm, followed by the allowable deviation in the Z direction, which is ±18 μm, and the allowable deviation in the X direction, which is 300 μm. If the power reduction is caused by the translation of the focusing mirror, it can be compensated through the translation of the optical fiber shown in Figure 8d. Meanwhile, the power compensation results in Figure 7c show that when the focusing mirror is shifted and the fiber is moved simultaneously for compensation, the highest coupling efficiency can be effectively ensured, which is of great significance for device packaging.

As shown in Figure 9, the vertical axis of the points in the figure represents the ratio of the power value to the maximum power when PBS and the rectangular prism are migrated in different directions. We empirically set a ratio of 98% or more to be the allowable tolerance.

As for the polarization combined beam path model, except for the allowable tolerance of the Z direction of PBS, which is about 2.3 mm, the allowable tolerance of the x and y directions of PBS and the x, y, and z directions of the rectangular prism are all within 1 mm. For x and y direction offset, the power change curves of PBS and the rectangular prism almost coincide, while in the z direction, P-polarized light is not affected by PBS’s z-direction offset, and s-polarized light can also focus because the rear focusing mirror size is large enough, so there will be a large tolerance. These data can be seen in Figure 9a. The rotationally polarized combined beam path model’s allowable deviation is similar to that of the polarization combined beam path model.

When considering the influence of angle deflection on system efficiency, we can see from Figure 9b that for the polarization combined beam path model, when the angle changes only a little, the system power changes a lot. PBS and right-angle prism have similar sensitivity to angle deflection, and the curves generally coincide. For the tx and tz angles, when the angle is ±0.06°, its power can be maintained at more than 98% of the highest power. However, the change in the ty angle has a greater influence on the power, and the allowable angle offset is only positive or negative 0.03°. These phenomena are improved in the rotationally polarized combined beam path model. Its tx, ty, and tz angle tolerances are ±0.09°, ±0.07°, and ±0.09°, respectively.

The reason why the system power is sensitive to the angle change may be that the angle between the incident light and the PBS intermediate membrane changes, resulting in a significant reduction in P wave transmittance and S wave reflectance, resulting in the beam not being able to pass through PBS normally. Even if the beam passes through PBS, small angle changes in rectangular prisms and PBS prisms may result in a certain exit angle that cannot be focused through the focusing mirror and thus cannot enter the fiber. Therefore, when installing PBS and rectangular prisms, it is very important to ensure that the angle is within a certain range to guarantee power. At the end of the whole model, the angle deviation of the polarization beam-combining system has such a great effect that the angle accuracy of the front prism must be guaranteed.

Since the rotationally polarized combined beam path model has a shorter path relative to the polarized combined beam path model, the residual divergence angle has relatively little effect on it, so it can be deviated by a relatively large angle.

## 4. Conclusions

In this paper, we proposed a rotating polarization high power, high spot homogenization optical coupling model based on the ray-tracing principle. Compared with the conventional optical coupling model, the model achieved a coupling efficiency of 89.04% and a numerical evaluation of 0.511 for spot uniformity. Maintaining a high-efficiency angle allows deviation and also increases the high uniformity of the spot distribution, avoids damage to the fiber caused by concentrating the energy at one point on the fiber surface, and is more conducive to subsequent spot focusing the effect by 50%. In future tasks, actual fiber-pumped lasers will be fabricated based on the optimized model for the commercial application of the devices.

## Figures and Tables

**Table 1:** Key parameters after beam collimation.

	ω (mm)	Φ (mrad)	ωBPP (mm·mrad)
Fast axis	0.305	1.94	0.592
Slow axis	1.655	4.98	8.242

**Table 2:** Reflectivity of optical elements.

Optical Elements	Reflectance/Transmittance	Size (mm)
FAC	0.2%	0.3 × 4 × 0.53
SAC	0.2%	2 × 0.8 × 0.6
Reflector	99.5%	3 × 1 × 0.2
HWP	0.2%	\
Rectangular prism	99.8%	2 × 2 × 2
PBS	P-polarized −90%, S-polarized −99.5%	\
Focusing mirror	0.5%	4 (Diameter) × 4

**Table 3:** Comparison of power and spot uniformity of the three optical path models.

Model	Non-Polarized Model	Polarization Combined Beam Path Model	Rotationally Polarized Combined Beam Path Model
Number of light sources	10	20	20
Spot uniformity	0.265	0.412	0.511
Power before focusing mirror (W)	95.53	183.44	184.94
Total power (in fiber)	91.06	177.22	178.08

## Data Availability

Data is unavailable due to privacy.
